# Thromboelastometry diagnosed Acute Traumatic Coagulopathy (ATC) identifies trauma patients with poor clinical outcomes better than Disseminated Intravascular Coagulation (DIC) scoring

**DOI:** 10.1186/1757-7241-21-S1-S30

**Published:** 2013-05-28

**Authors:** D Frith, M Lima-Baptista, R Davenport, C Rourke, J Manson, H De-Ath, S Khan, I Raza, K Brohi

**Affiliations:** 1 Trauma Sciences, Blizard Institute, Queen Mary University of London, London, UK

## 

Acute Traumatic Coagulopathy and Disseminated Intravascular coagulation are two definitions currently used to describe the endogenous hemostatic impairment that occurs early after injury. They have separate diagnostic criteria and different putative pathophysiology. The aim of this study was to determine which system better identifies trauma patients with high morbidity and mortality.

A prospective observational cohort study was performed at a level 1 trauma center. Blood was collected on arrival in the emergency department (ED) from all adult trauma patients who met the local criteria for full trauma team activation. Exclusion criteria included ED arrival >2 hours after injury or >2000mL of fluid before arrival. Blood was analysed by routine laboratory coagulation tests, rotational thromboelastometry and ELISA. Contingency analysis was performed by Fishers exact test.

Four hundred and thirty patients were included in the study. Seventy-six (18%) attended with ATC, as defined by EXTEM CA5 ţ 35mm. Laboratory coagulation results for DIC scoring were available at a median of 78 (62-103) mins. Eighteen (4%, p<0.001) met criteria for DIC on arrival using the amended ISTH scoring system. Of note, 86% of patients had elevated d-dimers without any scoring abnormality of prothrombin time, fibrinogen or platelets.

Overall, 115 patients required a blood transfusion within the first 12 hours. Thirty-seven (32%) of these met RoTEM criteria for ATC on admission but only fourteen (12%) had DIC (p<0.001). Similarly, 106 patients experienced a septic episode; 26% had ATC on admission but only 4% had DIC (p<0.001). Forty-seven patients died, 45% of whom had ATC on admission compared with 32% that could be diagnosed with DIC (p=0.2).

Although the pathophysiology of ATC remains to be fully elucidated, this study indicates that RoTEM diagnosis is more sensitive and clinically useful than DIC scoring criteria for the early identification of trauma patients with poor outcomes.

